# Prophylactic salpingectomy for prevention of ovarian cancer at the time of elective laparoscopic cholecystectomy

**DOI:** 10.1002/bjs.11419

**Published:** 2020-03-04

**Authors:** G. Tomasch, M. Lemmerer, S. Oswald, S. Uranitsch, C. Schauer, A.‐M. Schütz, B. Bliem, A. Berger, P. F. J. Lang, G. Rosanelli, F. Ronaghi, J. Tschmelitsch, S. F. Lax, S. Uranues, K. Tamussino

**Affiliations:** ^1^ Department of Obstetrics and Gynaecology Medical University of Graz Graz Austria; ^2^ Department of Surgery Medical University of Graz Graz Austria; ^3^ Department of Surgery Krankenhaus der Barmherzigen Brüder Graz Graz Austria; ^4^ Department of Gynaecology Krankenhaus der Barmherzigen Brüder Graz Graz Austria; ^5^ Department of Surgery Krankenhaus der Elisabethinen Graz Graz Austria; ^6^ Department of Obstetrics and Gynaecology Krankenhaus der Barmherzigen Brüder St Veit an der Glan Graz Austria; ^7^ Department of Surgery Krankenhaus der Barmherzigen Brüder St Veit an der Glan Graz Austria; ^8^ Department of Pathology Landeskrankenhaus Graz II Graz Austria

## Abstract

**Background:**

Most serous ovarian cancers are now understood to originate in the fallopian tubes. Removing the tubes (salpingectomy) likely reduces the risk of developing high‐grade serous ovarian cancer. Numerous gynaecological societies now recommend prophylactic (or opportunistic) salpingectomy at the time of gynaecological surgery in appropriate women, and this is widely done. Salpingectomy at the time of non‐gynaecological surgery has not been explored and may present an opportunity for primary prevention of ovarian cancer.

**Methods:**

This study investigated whether prophylactic salpingectomy with the intention of reducing the risk of developing ovarian cancer would be accepted and could be accomplished at the time of elective laparoscopic cholecystectomy. Women aged at least 45 years scheduled for elective laparoscopic cholecystectomy were recruited. They were counselled and offered prophylactic bilateral salpingectomy at the time of cholecystectomy. Outcome measures were rate of accomplishment of salpingectomy, time and procedural steps needed for salpingectomy, and complications.

**Results:**

A total of 105 patients were included in the study. The rate of acceptance of salpingectomy was approximately 60 per cent. Salpingectomy was performed in 98 of 105 laparoscopic cholecystectomies (93·3 per cent) and not accomplished because of poor visibility or adhesions in seven (6·7 per cent). Median additional operating time was 13 (range 4–45) min. There were no complications attributable to salpingectomy. One patient presented with ovarian cancer 28 months after prophylactic salpingectomy; histological re‐evaluation of the tubes showed a previously undetected, focal serous tubal intraepithelial carcinoma.

**Conclusion:**

Prophylactic salpingectomy can be done during elective laparoscopic cholecystectomy.

## Graphical abstract

Serous ovarian cancer is now understood to arise in the fallopian tubes. Prophylactic salpingectomy to reduce the risk of ovarian cancer is widely recommended at gynaecological surgery in appropriate women. This article explores prophylactic salpingectomy at the time of non‐gynaecological surgery.

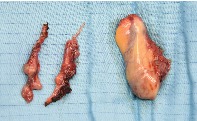

No harm, benefit still unclear

## Abstract (es)

**Antecedentes:**

La mayoría de carcinomas serosos de ovario se originan en las trompas de Falopio. La exéresis de las trompas (salpingectomía) probablemente reduce el riesgo de desarrollar un carcinoma seroso ovárico de alto grado. Numerosas sociedades ginecológicas recomiendan efectuar una salpingectomía profiláctica (u oportunista) en el momento de una cirugía ginecológica en determinadas mujeres, y esta conducta está ampliamente difundida. Sin embargo, no se ha analizado la realización de la salpingectomía durante cirugías no ginecológicas como forma de prevención primaria del carcinoma ovárico.

**Métodos:**

Determinar si la salpingectomía profiláctica con intención de reducir el riesgo de desarrollar cáncer de ovario sería aceptada y podría llevarse a cabo durante una colecistectomía laparoscópica electiva. Se reclutaron mujeres ≥ 45 años de edad programadas para colecistectomía laparoscópica electiva. A todas ellas se les aconsejó y ofreció la realización de una salpingectomía bilateral profiláctica en el momento de su colecistectomía. Las variables analizadas fueron la tasa de realización de la salpingectomía, la duración y las etapas quirúrgicos para efectuar este procedimiento, y las complicaciones.

**Resultados:**

La aceptación de la salpingectomía fue aproximadamente del 60%. La salpingectomía se realizó en 98 de 105 colecistectomías laparoscópicas (93%) y no se pudo realizar en 7 pacientes (7%) por escasa visibilidad o adherencias. La mediana del tiempo quirúrgico adicional fue de 13 (rango 4‐45) minutos. No hubo complicaciones atribuibles a la salpingectomía. Una paciente presentó cáncer de ovario 28 meses después de la salpingectomía profiláctica; la reevaluación histológica de las trompas mostró un carcinoma intraepitelial seroso focal tubárico (*serous tubal intraepithelial carcinoma*, STIC) no detectado previamente.

**Conclusión:**

La salpingectomía profiláctica se puede realizar durante la colecistectomía laparoscópica electiva.

## Introduction

Ovarian cancer is the leading cause of death among gynaecological malignancies and the fifth most common cause of cancer death among women in developed countries^1^. Despite aggressive surgical efforts and improved systemic therapies, progress against this disease has been slow^1^. There is an unmet need for effective primary or secondary prevention^2^, ^3^.

Most serous ovarian cancers are now recognized to originate in the fimbriae of the fallopian tubes^4^, ^5^, ^6^, ^7^, ^8^. Serous tubal intraepithelial carcinomas (STICs) were first described in 2001 by a Dutch group^9^ in women with *BRCA* mutations undergoing prophylactic removal of the tubes and ovaries. STICs are now considered precursors of high‐grade serous ovarian cancer, the most common histological subtype of the disease^4^, ^5^, ^6^, ^7^, ^8^. Accordingly, epidemiological studies^10^, ^11^, ^12^, ^13^, ^14^ have shown lower rates of ovarian cancer in women with a history of tubal sterilization.

The recognition that high‐grade serous ovarian cancer arises in the fallopian tubes, and that salpingectomy is associated with a reduced risk of developing ovarian cancer, has led to recommendations for prophylactic salpingectomy (opportunistic or risk‐reducing salpingectomy) at the time of benign gynaecological surgery, sterilization or caesarean section. Professional gynaecological societies, beginning with the Society of Gynecologic Oncology of Canada in 2011^15^, and including the Royal College of Obstetricians and Gynaecologists^16^, the Royal Australian and New Zealand College of Obstetricians and Gynaecologists^17^, the American College of Obstetricians and Gynecologists^18^ and the Austrian Society of Obstetrics and Gynaecology^19^, have issued statements to the effect that salpingectomy should be considered at the time of pelvic surgery in appropriate women^20^. Salpingectomy at the time of gynaecological procedures requires little additional operating time, and is not associated with prolonged hospital stay, surgical complications or readmission^21^, ^22^, or long‐term sequelae. In the USA, Dilley and colleagues^23^ calculated that salpingectomy at the time of hysterectomy for prevention of ovarian cancer is both cost‐effective and cost saving. Accordingly, prophylactic salpingectomy at the time of gynaecological surgery is now performed widely and routinely in many areas of the world^20^, ^21^, ^22^, ^23^, ^24^, ^25^, ^26^, ^27^.

Cholecystectomy for benign gallbladder disease is commonly performed in women and the large majority of these procedures are done laparoscopically^28^, ^29^, ^30^. Thus, elective laparoscopic cholecystectomy presents a potential opportunity for incidental salpingectomy (and sterilization) in appropriate women.

A series of semistructured interviews in women scheduled for elective laparoscopic cholecystectomy indicated that a majority were open to the idea of opportunistic salpingectomy^31^. The aim of the present study was to evaluate the feasibility and short‐term complications of prophylactic salpingectomy in women aged 45 years or older undergoing non‐emergency laparoscopic cholecystectomy for benign indications.

## Methods

The study protocol was approved by the ethics committees of the Medical University of Graz and the participating institutions, and was registered with http://clinicaltrials.gov (NCT03171467). The aim was to recruit 100 women aged at least 45 years scheduled for elective laparoscopic cholecystectomy for benign gallbladder disease. Further inclusion criteria were: childbearing completed; knowledge of German sufficient for detailed consent; and ability to complete questionnaires. Excluded were: women aged less than 45 years; desire to preserve fertility; emergency procedures; extensive previous surgery with probable difficult access to the pelvis; insufficient command of German; and a family history suggesting a *BRCA* mutation. The time between offering salpingectomy and scheduled cholecystectomy was stipulated as at least 7 days.

Women who met the inclusion criteria were informed of the possibility and rationale for elective salpingectomy at least 7 days before planned surgery. Consenting patients were asked to complete a questionnaire developed previously^31^. Counselling regarding prophylactic salpingectomy was provided by a surgeon, a gynaecologist or both. Counselling included a detailed explanation of the tubes and what they do, that salpingectomy entails removal of the tubes with preservation of the ovaries, the rationale for prophylactic salpingectomy (prevention of ovarian cancer), that removal of the tubes precludes further natural conception, that it does not affect the hormonal cycle, and that port repositioning (additional port sites) might be required. Patients were informed that salpingectomy might not be possible for reasons such as presence of adhesions. All patients provided signed informed consent.

Patients did not incur costs and physicians did not receive fees for additional prophylactic salpingectomy.

Laparoscopic cholecystectomy was performed according to institutional preference, generally with a 10‐mm umbilical port and ancillary ports of 3 or 5 mm. The protocol stipulated abandoning salpingectomy if not readily feasible, for example if adhesions blocked easy access to the tubes.

Outcomes were: rate of accomplishment of prophylactic salpingectomy (completed/attempted salpingectomies); time needed for salpingectomy (including time waiting for a gynaecological surgeon); intraoperative or postoperative problems or complications attributable to salpingectomy; and 30‐day readmission rate. Also recorded were: whether ports were repositioned for salpingectomy; whether preexisting scars could be used; whether additional instruments were used for salpingectomy; and whether salpingectomy was done by surgeons, gynaecologists or both.

Pathological processing of the tubes was at the discretion of the pathologists; an extended protocol (sectioning and extensively examining the fimbria, SEE‐FIM^32^) was not stipulated. The trial was not designed for follow‐up beyond 30 days.

## Results

A total of 105 patients, of mean age 55 (range 42–79) years and median parity 2 (range 0–4), scheduled for elective laparoscopic cholecystectomy consented to concomitant prophylactic salpingectomy. Six centres participated. Two centres entered 52 and 29 patients, and four entered ten or fewer patients. The rate of acceptance of attempted salpingectomy was 62 per cent at the three centres that entered the largest number of patients. Indications for cholecystectomy were cholelithiasis (98), gallbladder dyskinesia (6) or both (1).

**Figure 1:**
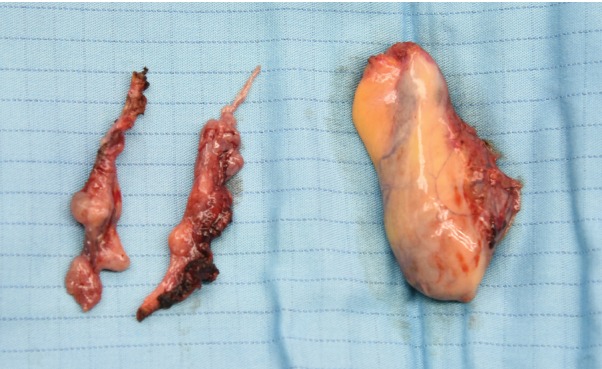
Surgical specimens obtained at laparoscopic cholecystectomy and prophylactic salpingectomy

Salpingectomy was completed as planned in 98 (93·3 per cent) of the 105 patients (*Fig*. 1). Salpingectomy was not possible in seven patients as adhesions precluded easy access to, or visualization of, the tubes. Age and BMI did not differ between patients who did and those who did not undergo prophylactic salpingectomy. Malignancy was not detected in any gallbladders or fallopian tubes.

Surgical data are shown in *Table* 1. The median additional time required for salpingectomy was 13 min. There were no intraoperative or postoperative complications attributable to salpingectomy. After surgery, one patient developed pancreatitis and stayed in hospital for 13 days. One patient was readmitted to hospital 6 days after surgery with abdominal pain that resolved the next day.

**Table 1:** Surgical data for 105 patients in whom salpingectomy was attempted at the time of laparoscopic cholecystectomy

Laparoscopic cholecystectomy	No. of patients (*n* = 105)
**No. of ports**	
Multiple	102
Single	3
**Instrument used for cholecystectomy**	
Monopolar coagulation	51
Bipolar coagulation	49
Vessel sealing	2
Ultrasonic energy	3
**Different modality used for salpingectomy or new device needed**	32
**Total duration of operation (min)** *	67 (2–137)
**Operating time for salpingectomy only (min)** *	13 (4–45)
**No. of trocars repositioned**	
0	89
1	13
2	1
3	0
Unclear	2
**Previous scars used (e.g. appendicectomy)**	3
**No. of new trocars used**	
0	83
1	15
2	1
3	0
Unclear	6
**Salpingectomy completed as planned**	
Yes	98
No	7
**Surgeon performing salpingectomy**	
General surgeon	79
Gynaecologist	19
Both	7
**Duration of postoperative hospital stay (days)** *	2 (1–13)

* Values are median (range).

**Figure 2:**
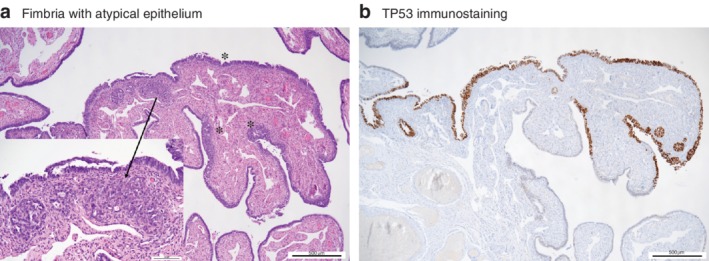
Images of serous tubal intraepithelial carcinoma Serous tubal intraepithelial carcinoma in a patient who developed peritoneal carcinomatosis of a high‐grade serous carcinoma 28 months after prophylactic salpingectomy. **a** The fimbria is partially covered by markedly atypical epithelium (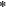), which is highlighted by a mutant immunoreactive pattern for TP53. An area of approximately 0·5 mm is suspicious for early stromal invasion (**a**, insert, marked by arrow) but could not be confirmed at deeper levels (**b**). (**a** Haematoxylin and eosin stain; **b** 3,3′‐diaminobenzidine immunostain with haematoxylin and eosin counterstain.)

Although follow‐up was until 30 days after surgery, one 57‐year‐old woman presented with ascites and peritoneal carcinomatosis owing to high‐grade serous carcinoma 28 months after surgery. The initial pathology report had described normal tubes; re‐evaluation of the slides showed a small, focal STIC (*Fig*. 2).

## Discussion

A previous study^31^ showed that women aged 45 years or older scheduled for elective laparoscopic cholecystectomy were open to the idea of prophylactic salpingectomy with the intention of preventing ovarian cancer. In the present study, approximately 60 per cent of women who were approached consented to having prophylactic salpingectomy. The study showed that prophylactic salpingectomy is feasible at the time of laparoscopic cholecystectomy, with salpingectomy performed in 98 of 105 patients. Salpingectomy was not associated with complications and added just a median of 13 min to the operating time, consistent with a study^33^ that reported an additional 14 min during gynaecological surgery.

The protocol emphasized that salpingectomy was to be abandoned if the tubes were not easily accessible. This was the case in seven procedures owing to adhesions. Clearly, a benefit of salpingectomy for cancer prevention would quickly be negated if there was an appreciable rate of complications associated with the procedure. A number of studies^21^, ^22^, ^23^, ^24^, ^25^ in the gynaecological literature have indicated that prophylactic salpingectomy is feasible, safe and cost‐effective at the time of gynaecological surgery. Some judgement is required regarding when not to pursue salpingectomy, for instance in a woman with a history of extensive pelvic surgery or diverticulitis.

A 57‐year‐old woman in this study presented with carcinomatosis owing to serous cancer 28 months after prophylactic salpingectomy; she had a STIC that was not seen at routine evaluation of the tubes. The present study was not set up to assess the prevalence of STIC lesions or cancer, and a SEE‐FIM protocol^32^ is not used routinely for grossly normal tubes removed from women with no increased risk of ovarian cancer (as opposed to specimens from women with a *BRCA* mutation). A recent Canadian study^34^ that used the SEE‐FIM protocol found STIC in eight of 9392 women (less than 0·1 per cent) with benign diagnoses who had a normal risk of ovarian cancer.

Implementing prophylactic salpingectomy at the time of non‐gynaecological surgery would pose a number of challenges. Careful and thorough informed consent is required. The consent process needs to cover the anatomy and function of the tubes and ovaries, the rationale for the procedure, and the consequences of removing the tubes while preserving the ovaries. This raises reproductive and endocrine issues that are outside the scope of other consent processes in general surgery. In the public healthcare system in Austria, within which the patients in this series received care, physicians are salaried and patients are not billed. However, billing issues may arise in other systems. General surgeons may not feel comfortable offering or doing the procedure, or transferring it to a gynaecology colleague (or waiting for a gynaecologist to come to the operating room). In clinical practice it may be difficult to coordinate counselling, consent and the surgery itself between general surgeons and gynaecologists. The unit that recruited most patients in the present series was at an institution with no in‐house gynaecologists; general surgeons managed the counselling, consent and the salpingectomy itself.

Prophylactic salpingectomy has become routine at the time of gynaecological surgery in appropriate women in many countries^20^. The present results suggest that prophylactic salpingectomy can be done in women undergoing non‐gynaecological surgery. However, the results may not be generalizable to other healthcare settings and systems. Potentially, salpingectomy could be accomplished during many other laparoscopic or other minimally invasive procedures. This would require non‐gynaecologists to inform patients scheduled for surgery of risks and benefits of an additional procedure on the genital tract. Implementing this will be an exercise in bridging surgical silos^35^. To date there are no prospective data confirming that opportunistic salpingectomy reduces cases of and deaths from ovarian cancer.
